# Towards defining reference materials for measuring extracellular vesicle refractive index, epitope abundance, size and concentration

**DOI:** 10.1080/20013078.2020.1816641

**Published:** 2020-09-24

**Authors:** Joshua A. Welsh, Edwin van der Pol, Britta A. Bettin, David R. F. Carter, An Hendrix, Metka Lenassi, Marc-André Langlois, Alicia Llorente, Arthur S. van de Nes, Rienk Nieuwland, Vera Tang, Lili Wang, Kenneth W. Witwer, Jennifer C. Jones

**Affiliations:** a Translational Nanobiology Section, Laboratory of Pathology, National Cancer Institute, National Institutes of Health, Bethesda, USA; b Vesicle Observation Center, Amsterdam UMC, Location AMC, University of Amsterdam, Amsterdam, The Netherlands; c Biomedical Engineering & Physics, Amsterdam UMC, University of Amsterdam, Amsterdam, The Netherlands; d Laboratory of Experimental Clinical Chemistry, Department of Clinical Chemistry, Amsterdam UMC, Location AMC, University of Amsterdam, Amsterdam, The Netherlands; e Department of Biological and Medical Sciences, Faculty of Health and Life Sciences, Oxford Brookes University, Oxford, UK; f Laboratory of Experimental Cancer Research, Department of Human Structure and Repair, Ghent University Hospital, Ghent, Belgium; g Cancer Research Institute Ghent, Ghent, Belgium; h Institute of Biochemistry, Faculty of Medicine, University of Ljubljana, Ljubljana, Slovenia; i University of Ottawa Flow Cytometry and Virometry Core Facility, Ottawa, Canada; j Department of Biochemistry, Microbiology and Immunology, Faculty of Medicine, University of Ottawa, Ottawa, Canada; k Ottawa Center for Infection, Immunity and Inflammation, Ottawa, Canada; l Department of Molecular Cell Biology, Institute for Cancer Research, Oslo University Hospital, Norway; m VSL Dutch Metrology Institute, Delft, The Netherlands; n National Institute of Standards and Technology (NIST), Gaithersburg, MD, USA; o Departments of Molecular and Comparative Pathobiology and Neurology, The Johns Hopkins University School of Medicine, Baltimore, MD, USA

**Keywords:** Calibration, exosomes, extracellular vesicles, microvesicles, optical analysis, reference materials, standardization, quality control, validation

## Abstract

Accurate characterization of extracellular vesicles (EVs) is critical to explore their diagnostic and therapeutic applications. As the EV research field has developed, so too have the techniques used to characterize them. The development of reference materials are required for the standardization of these techniques. This work, initiated from the ISEV 2017 Biomarker Workshop in Birmingham, UK, and with further discussion during the ISEV 2019 Standardization Workshop in Ghent, Belgium, sets out to elucidate which reference materials are required and which are currently available to standardize commonly used analysis platforms for characterizing EV refractive index, epitope abundance, size and concentration. Due to their predominant use among EV researchers, a particular focus is placed on the optical methods nanoparticle tracking analysis and flow cytometry.

## Introduction

“Extracellular vesicles” is an umbrella term for lipid bilayer-delimited particles derived from cells through a number of pathways; these include exosomes and ectosomes [1]. The connection of EVs to many aspects of human health and disease surged a global interest in the development of EV-based biomarkers and therapeutics [2]. The use of EVs requires techniques which are able to reliably characterize their attributes including refractive index, epitope abundance, size and concentration. EVs can be studied by bulk and single-particle techniques. A bulk technique measures one or more properties of a bulk EV population, e.g. enzyme-linked immunosorbent assay (ELISA), Western blot, total protein/lipid/nucleic acid concentration or bead-based flow cytometry. Single-particle techniques characterize EVs one-by-one. Examples of single-particle techniques are flow cytometry, nanoparticle tracking analysis (NTA, a commercialized name of single-particle tracking (SPT), electron microscopy (EM) and resistive pulse sensing (RPS). Bulk techniques are often scalable, sensitive and therefore compliant with routine clinical applications. For example, the first widely used screening test for human immunodeficiency virus (HIV) was based on ELISA [3]. Despite these benefits, the use of bulk techniques has only limited abilities to improve understanding of disease-specific EV subsets due to the heterogeneity of EVs in of many body fluids. Bodily fluids often contain many different particles with overlapping size and physical properties, such as lipoprotein particles, protein complexes, small platelets and EVs from many cell types. When applied to putative disease-specific EV subsets, therefore, interpretation of bulk techniques may rely heavily on the purity of the EV preparation. For example, a Western blot positive for an EV marker may be informative for a highly purified EV population, but not for a neat biological fluid, where the signal could come from soluble protein. In contrast, single-particle techniques have the potential to identify single EVs and differentiate between EV subsets (EVs with common molecular profile or cargo) and other non-EV particles. If the technique is high-throughput and allows sufficiently multiplexed phenotypic characterization, it could even obviate the need for separation, a particularly important consideration for clinical applications.

The detection of single EVs in body fluids is challenging because EVs are: (1) heterogeneous in size, with the majority having a diameter <200 nm; (2) are also heterogeneous in composition, biogenesis and origin; (3) have a low (<1.42 for EVs >200 nm) refractive index (RI); and (4) often co-exist with non-EV components that overlap in biochemical composition and/or physical properties [4]. The diameter distribution of EVs in normal human plasma and urine has been shown to range between 50 nm and >1,000 nm [5,6]. Because instruments detecting single EVs, such as flow cytometers, differ in sensitivity and because only a fraction of the EVs exceeds the detection threshold, minute differences in the lower limit of detection will strongly affect the measured concentration of EVs [6]. Understanding the performance strengths and limitations of single EV characterization techniques is crucial to generate reliable and reproducible data and can also help to identify approaches to improve these analysis techniques and assays [7].

To become clinically relevant, EV measurements must be standardized. To this end, standard reference materials and reference procedures require development. There is a growing awareness that the reliability and reproducibility of EV measurements need to improve. These efforts have materialized in a number of formats including publication of “minimal requirements” (MISEV2014, MISEV2018), improved recording of experimental parameters in “EV-TRACK” [1,7–9], and standardization studies on EV concentration measurements by tunable RPS [10], flow cytometry [11,12], NTA [13] and functional coagulation assays [14]. Recently, the International Societies of Extracellular Vesicles (ISEV), Advancement of Cytometry (ISAC), and Thrombosis and Haemostasis (ISTH) EV flow cytometry working group published a position paper, delineating a “minimum information for the reporting of an EV flow cytometry experiment” (MIFlowCyt-EV) standard reporting framework, that will increase the transparency and reproducibility of EV flow cytometry experiment protocols and reporting [15].

While the need for reference materials is increasingly recognized, the nomenclature and purpose of reference materials within the EV field are currently poorly defined, with some nomenclature commonly misused. Here, we focus on understanding what is meant by a reference material, what types of reference material are required by the EV field. This analysis highlights how reference materials and EV samples should be characterized and reported. The majority of single EV measurements are currently performed using optical characterization methods such as NTA and flow cytometry [16]. We will therefore focus on standardization of these analysis methods and then compare these with non-optical measurement techniques.

## Assessing common EV characterization techniques

A range of analysis techniques has been used to characterize EVs. Table 1 provides a comparison of popular EV analysis techniques, indicating their ability to provide measurements of diameter, immunophenotyping data, concentration, refractive index, single-particle detection, detect all individual EVs, have a derivable sensitivity limit, and achieve a large sampling of particles (>10,000 events). As one of the first characteristics of an EV analysis technique is assessing whether it is detecting a signal from single or multiple (bulk) particles, techniques are split into these two respective categories.

**Table 1.** **Comparison of highly reported EV characterization methods**. For diameter, immunophenotyping, concentration and refractive index ticks depict whether the instrument provides or has been demonstrated to prove a particular measurement metric. For diameter, immunophenotyping, concentration and refractive index, crosses indicate the instrument does not, is not able to, or has not been demonstrated in published literature to provide a particular element at the time of writing this review. EV population detection refers to the ability of the technique to be feasibly capable of detecting sizes of 30–1000 nm We define high-throughput as being able to feasibly analyse 10,000 or more events per sample in <3 minutes. SP-IRIS refers to single particle interferometric reflectance image sensing with optional fluorescence detection.

### Diameter distribution determination

Single-particle methods are needed to generate accurate size distributions. For this reason, NTA, flow cytometry and electron microscopy have been widely used for EV diameter distribution reporting [16]. Newer methods such as resistive pulse sensing, super-resolution microscopy and interferometric reflectance imaging sensing (IRIS) are also beginning to be utilized [17,18]. Bulk methods such as dynamic light scattering (DLS) are increasingly recognized as insufficient in the field and are used less frequently, because bulk methods can be prone to biases arising from the heterogeneity of EV samples and skewed particle size distribution.

### Molecular phenotyping

Bulk analysis techniques, such as Western blots, ELISAs, mass spectrometry, and sequencing, and bead capture assays, have been widely used and instrumental in the field to date, associating EV phenotype (molecular cargo) with function. However, bulk analysis methods cannot convey if a particular analyte is in or on all EVs or just a subset, reveal the distribution of markers within a positive subset, or identify the size distribution or concentration of the positive subset. Bulk techniques therefore lack the ability to characterize the heterogeneity of the EV population, which could be seen as critical for some of their intended uses in clinical chemistry. For this reason, there is a strong impetus to develop single-particle analysis techniques, and an increasing number of platforms have become available. As seen in Table 1, only electron microscopy and super-resolution microscopy are capable of phenotyping single EVs of the smallest diameter. Though these methods are specialized and low-throughput (time-intensive) and can analyse only a small portion of the population, possibly neglecting low abundance particles such as large EVs. High-throughput methods are therefore desirable for single-particle phenotyping. NTA can technically be used for high-throughput fluorescence-based phenotyping, but low detection sensitivity and fluorophore bleaching have limited its application. Flow cytometry is another high-throughput possibility, but a lack of minimum procedural and reporting guidelines for single EV flow cytometry, combined with variable equipment sensitivities, settings and staining methodologies, has led to a general lack of reproducible data. This has only recently been address in the form of the MIFlowCyt-EV framework [15].

### Concentration determination

The determination of concentration is a multifactorial measurement as it quantifies the number of particles within a set volume. How well the measurement signal is being differentiated from background, e.g. are all of the EVs detectable, and the ability to accurately determine the analysed volume both play a role in accurate concentration determination. If a technique is unable to detect all particles within a population, an absolute EV concentration metric is not possible as particles below the limit of detection are not being counted. In instances where all EVs are not detected, the correct method for reporting of a particle concentration measurement is the concentration of detected particles within a limit of detection, i.e. 3 × 10^7^ particles mL^−1^, limit of detection = 157 nm, that can be reproduced.

We define the lower limit of detection (limit of sensitivity), as the threshold at which a signal (such as light scatter for NTA) from a particle of given size can no longer be discriminated from the background. The limit of sensitivity can be described in numerous ways, with some easier to quantitate than others. In the above example with NTA, the limit of detection could be reported in terms of the number of photons needed to resolve a signal from background, or as the diameter of a particle with a given refractive index. Despite this, it is also one of the few single-particle measurement techniques that also does not have a definable sensitivity limit with regard to light scatter intensity or fluorescence intensity in standard units.

### Sensitivity versus resolution

**Figure 1.**
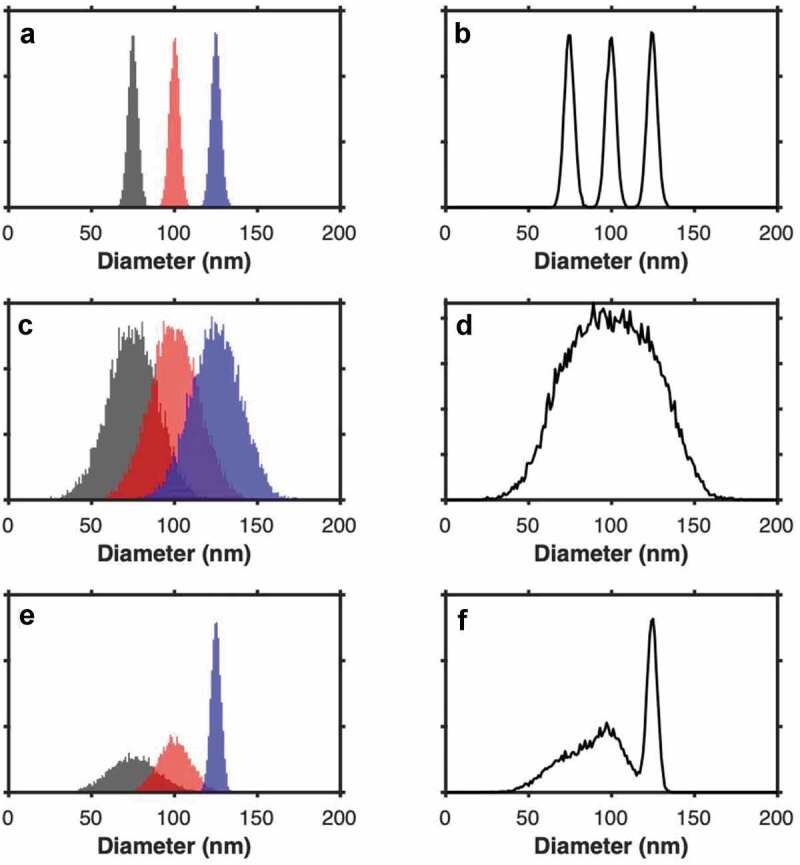
**The importance of resolution**. a) demonstrated the detection of particles with a consistently high resolution (µ = 75, 100, 125 nm, *σ* = 3, 3, 3), b) shows the cumulative diameter distribution of particles from plot a. c) demonstrated the detection of particles with a consistently low resolution (µ = 75, 100, 125 nm, *σ *= 15, 15, 15), d) shows the cumulative diameter distribution of particles from plot c. e) demonstrated the detection of particles with a typical detection technique resolution, whereby it decreases as the signal becomes smaller (µ = 75, 100, 125 nm, *σ *= 15, 10, 3), f) shows the cumulative diameter distribution of particles from plot e. All populations have 10,000 particles.

Distinguishing between “sensitivity” and “resolution” is important and these terms are often misused within the EV literature. In most cases when instruments other than microscopes are calimed to be “high-resolution”, in fact only their sensitivity is quantified. This is particularly apparent in flow cytometry with measurements such as scatter, where only the sensitivity can be quantified and no formal method of quantifying light scatter resolution currently exists. This is due to complex relationships between particle light scattering attributes and collection optics [19]. While “sensitivity” describes the ability to detect a signal, “resolution” describes the ability to distinguish one signal from another. Figure 1, depicts hypothetical results when three techniques are used to detect particles with diameters of 75, 100 and 125 nm: measured either as single populations (left column, Figure 1(a,c,e) or when mixed together (right column, Figure 1(b,d,f)) to approximate a heterogeneous mixture of EVs. In this example, each particle population contributes an equal number of events. Although each technique is sufficiently *sensitive* to detect the individual populations, *resolution* differs substantially. The method in Figure 1(a,b) has high resolution. Figure 1(c,d) shows a low-resolution method. The technique in Figure 1(e,f) loses resolution as particles (and their signal) become smaller. This latter pattern is typical of detection methods such as flow cytometry, NTA and RPS. Clearly, our perception of a population size distribution can be skewed if resolution is not taken into account.

### Current techniques: an overview

Each currently available platform for single EV characterization has strengths and weaknesses. NTA is widely used to estimate size and concentration [20,21]. NTA is also capable of phenotyping (when combined with affinity-linked fluorescence) and effective refractive index measurements [20,22–24]. However, it is unable to detect all EVs, and there is currently no published protocol to determine a lower limit of sensitivity in standard units for fluorescence or scatter [6,20]. This is currently also true for the combination of IRIS (interferometry) and fluorescence. RPS is also incapable of detecting all individual EVs, as the pores used for the measurement have discrete size ranges [6,25]. However, RPS can measure tens of thousands of events in a short period of time, and its measurement sensitivity can be deduced using currently available traceable size standards. Since RPS does not allow affinity-based phenotyping, EV size and concentration can be interpreted correctly only for populations well separated from potential co-isolates, such as lipoproteins in plasma. This is also the case for other techniques where affinity-based phenotyping is not or cannot be done. Currently, only single-EV flow cytometry combines abilities for sizing, concentration measurement, affinity-based phenotyping and high throughput with calibration into standard units to provide a limit of sensitivity for each parameter [26–29]. While not all techniques have a discernable limit of sensitivity that can be derived from a calibration and expressed in standard units, it is possible to perform indirect assessments of sensitivity using reference materials. It is also possible to track performance using quality controls. These are discussed in detail below.

## Background to reference materials

At present, many investigators in the EV field use the term “reference material” to refer to any material that assists with evaluation of reproducibility of a measurement. While “EV reference materials” have been a common theme of “what is needed” in the field, leading to many initiatives including a 2019 ISEV Workshop and Standardization Task Force dedicated to “EV Reference Materials”, most discussions of reference materials do not consider metrological first principles, including the need for traceable measurements. By traceable, we mean that the measurement result can be related to the relevant SI unit(s) through an unbroken chain of comparisons with known uncertainties. If all labs were to report their data in SI units, measurement results would become comparable. Consideration of the need for making traceable measurements highlights a more basic need in the field: to first develop standard reference materials for each of the parameters that will be measured, such as refractive index, epitope abundance, size and concentration. Because equipement in typical EV laboratories, such as flow cytometry, NTA and RPS, is not calibrated in a traceable manner, using the basic principles of metrology, the development of such reference materials requires knowledge and equipment from metrology institutes. In the next section, we will introduce metrology and the metrological meaning of the term “reference material”.

Metrology is the science of measurement and is regulated by the International Bureau of Weights and Measurements (BIPM) and ensures international unification of physical measurements and nomenclature between regulatory agencies. The BIPM operates under the exclusive supervision of the International Committee for Weights and Measurements (CIPM), which established the International System of (SI) Units in 1960. The SI unit, known as the metric system, is the international measurement standard. The SI unit is recognized in nearly 50 countries, with the CIPM disseminating and modifying the definition of SI units as technology progresses. The International Organization for Standards (ISO) is an independent, non-government organization that interacts with BIPM. ISO is the largest developer of international standards and provides common standards to more than 160 countries. Over 22,910 standards have been published to date. An example of an ISO standard is the definition of accuracy, ISO 5725–1:1994, “Accuracy (trueness and precision) of measurement methods and results”. National metrology institutes, such as the US National Institute for Standards and Technology (NIST), develop certified reference materials (Standard Reference Materials®) which are traceable to the SI unit using ISO standards.

**Figure 2.**
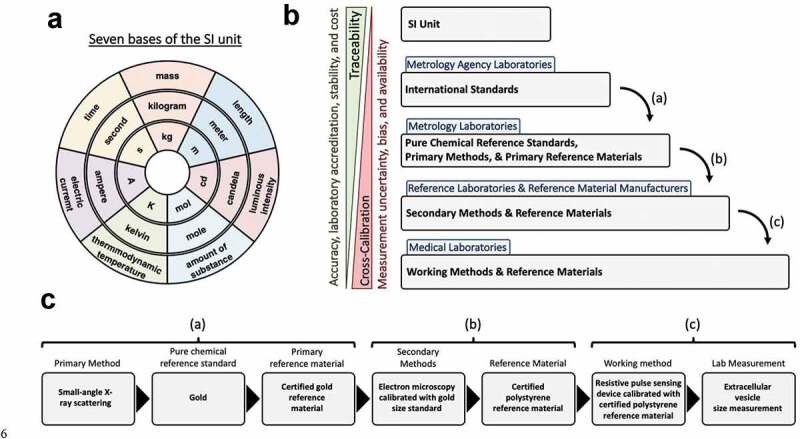
**Traceability to the SI unit**. a) shows the seven base units of the SI unit, the outer circle shows the base unit measurement, the middle circle shows the measurement unit, the inner circle shows the measurement unit symbol. b) Hierarchy of traceability from the working methods and reference materials to the SI unit. c) shows an example of an EV measurement using RPS back to the SI unit.

**Figure 3.**
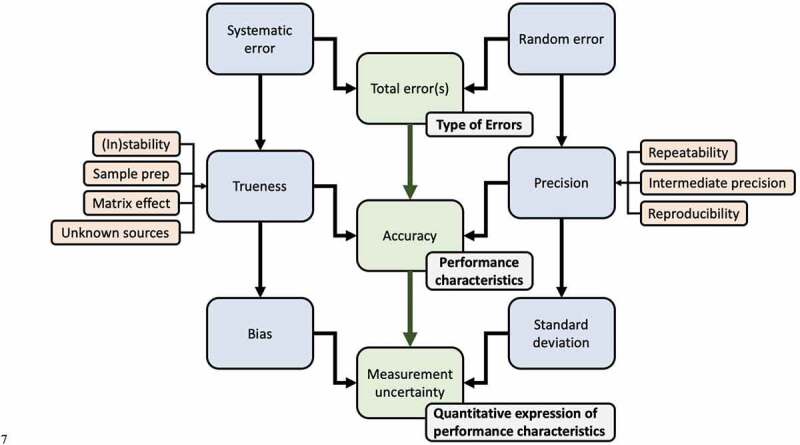
Parameters involved in characterizing a certified reference material.

The SI system is made up of seven base units that define 22 derived units with special names and symbols, Figure 2(a). The measurements performed using certified reference materials within laboratories can be traced back to the SI unit, Figure 2(b). An example is a gold size standard being made and characterized in comparison to an international size standard, Figure 2(c). This gold size standard is used by manufacturers of polystyrene size standards which are characterized in relation to the gold size standard by electron microscopy. These polystyrene size standards can then be bought commercially and used to calibrate laboratory instrumentation. The working methods and reference materials with the laboratory instrumentation are then used to characterize assays output, e.g. the size of an EV. As reference materials continue to be cross-calibrated, their accuracy and stability may decrease due to measurement uncertainity, Figure 2(b) [30]. This variation may originate from parameters such as the reproducibility of the calibration measurement, the accuracy of the algorithm required, or drift within measurements, Figure 3 [30].

In metrology, “Reference material” is a generic term that refers to any material that is sufficiently homogeneous and stable with respect to one or more properties, and which has been established to be fit for its intended use in a measurement process [31]. A “certified reference material” is characterized by a metrologically valid procedure for one or more of its properties and is accompanied by a certificate providing the values of the specified property, a statement of metrological traceability and associated uncertainty. Metrologically valid procedures for production and certification are outlined in ISO Guide 31, 34 and 35. Reference materials can have different applications, e.g. calibration, validation, quality control, etc. Each of these applications can require varying degrees of accuracy in their reference material characterization, e.g. international standards, certified reference materials, working reference materials.

**Figure 4.**
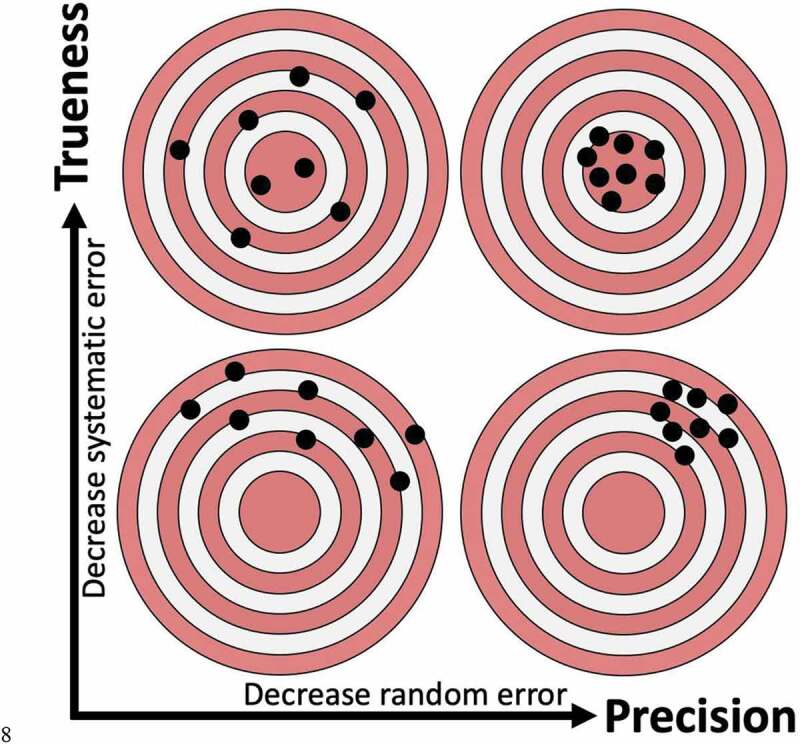
Visualizing the difference between trueness and precision.

For a reference material to be traceable back to an SI unit, the uncertainty of a measurement must be known, Figure 3. The uncertainty of a measurement is the quantification of doubt about the measurement and is based upon the standard deviation and bias of a measurement. The standard deviation describes the precision of a measurement due to random error. The measurement bias describes the trueness of a measurement, which can be affected by systematic error. “Error” in both systematic and random errors describes the difference between the measured value and the “true value” of the property being measured. The trueness and precision of a measurement, defined by ISO 5725, equate to the accuracy of a measurement, Figure 4. The terms accuracy and precision are frequently used within the literature with definitions that do not align with those defined by ISO 5725. In most cases, “precisions and accuracy” are referred to despite precision being a component of accuracy. A measurement whereby the result is low in systematic error but high in random error is considered high in trueness but lacking in precision. A measurement whereby the result is high in systematic error but low in random error is considered low in trueness but high in precision. Understanding these concepts is critical for the development of EV reference materials, assessing EV analysis equipment, and general investigation and characterization of EVs.

## Reference material categories for standardization of EV characterization techniques

The development and utilization of reference materials may fall into one or more of the following categories: *calibration, validation* and *quality Control* The need for certified vs uncertified reference materials for these needs will vary, as discussed below.

### Calibration

It is the conversion of an arbitrary unit scale to a scale in standard units. For example, converting the fluorescence intensity scale of a flow cytometer from arbitrary units to a scale of fluorophore number. Calibration requires well-characterized reference particles. Ideally, the calibration reference materials would also be certified, so as to give a traceable measurement back to SI units and limiting the potential for bias in calibration accuracy. The development of certified calibration reference particles is crucial for instrument measurement calibration, instrument sensitivity quantification and in turn instrumentation standardization and comparisons [29]. Calibration reference materials allow standardized reporting and consequently, validation of published EV studies between assays, instruments and laboratories. The development of accurate calibration reference materials also allows the development of standardized quality control reference materials.

Calibration reference materials do not necessarily need to mimic all characteristics of the downstream analysis particles, such as EVs. Materials such as polystyrene beads, hollow organosilica beads, liposomes, etc., could all be conceivably used as size standards, so long as the measurement technique sizing ability is based on physical size and not on other properties such as light scatter intensity (which would also depend on refractive index) [29]. Calibration reference materials for some parameters, such as fluorescence and light scatter, typically require a set of standards for multiple populations [19,32,33]. Other parameters, such as concentration, can be based on a single calibration reference material, though are improved with multiple populations across the detection range.

### Validation

It assesses an assay’s sensitivity and specificity by using a known sample, such as a positive or negative control. For example, a scale could be calibrated to diameter units using a 100 nm certified diameter standard. To validate the calibration of the scale, a 150 nm certified diameter standard could be analysed to check that the population appeared at 150 nm on the scale. An assay’s specificity for detecting CD41a-positive population could be validated by using a population of CD41a certified positive particles for a positive result. Inversely, the assay specificity for negative results could be validated using CD41a certified negative particles for a negative result. Validation reference materials appear to be the most sought-after within the EV field in order to test detection methods and assays [34–36]. These types of reference materials can be used as positive and negative controls within assays by having previously characterized properties, such as protein expression, concentration, diameter distribution, composition, etc. While in some cases calibration reference materials can also be used for assay validation, validation should not be carried out using the same reference material as used for calibration. For example, if an assay is calibrated with a 100 nm diameter bead, validating the measurement with the same 100 nm bead instead of an independent size standard could lead to measurement bias and mask an error in the calibration procedure. Despite great potential for the use of EV reference materials for assay validation, currently only uncertified EV reference materials are commercially available, Table 2. Uncertified reference materials can lack accuracy in characterization measurements, and depending on how they are used for their downstream application can lead to bias in sample characterization, e.g. using an incorrectly assigned size standard to calibrate a RPS device will lead to measurement inaccuracy of downstream sample diameter measurements. Many commercially available reference materials have not been rigorously quantified or otherwise characterized, and sometimes not at all. These types of reference materials are therefore not standard, and may not be traceable across different detection methods, and thus result in variable data across instrumentation. Their use as quality control reagents or assay development reagents on a single platform may, however, be useful.

**Table 2.** **Comparison of basic optical parameter characterization of commercially available reference materials**. Information was collated using manufacturer websites and product sheets that were openly available. It is possible further is known about these products, but that information is not freely/openly available at the time of writing this review. Ticks depict whether information is provided by the manufacturer. Asterisks highlight measurement types that require a limit of detection to be provided. The last row shows if a limit of detection has been provided for any of the relevant measurements. SAXS; small angle x-ray scattering.

The development of certified EV reference materials is currently impeded due to limitations in sensitivity and resolution of commercially available analysis techniques, Table 1. Most current commercially available techniques are not able to detect and/or phenotype the full range of the EV population as single particles and/or cannot provide a large enough sampling of the population to produce robust measurements. Furthermore, many of the techniques that can provide a higher sampling of the EV population, as seen in Table 1, cannot yet be calibrated to provide a quantitative sensitivity limit or traceable measurement. The measurement bias of these techniques can therefore not be accounted for, and the “product specification” metrics provided along with reference materials will likely be inaccurate.

***Quality control*** assesses the performance of an assay (“repeatability” and “reproducibility”) to verify that it produces consistent results over time. Repeatability assesses whether a measurement is consistent when performed: at the same location; using the same measurement procedure; by the same observer; using the same measuring instrument, used under the same conditions; and repeated over a short period of time. “Reproducibility” assesses whether measurements are consistent when conducted by different individuals, at different locations, and with different instruments. Quality control can be assessed intra-assay with replicate samples or inter-assay with calibration or validation reference materials. Quality control for all measurement techniques is essential and is best quantified in standard units, e.g. “the detection sensitivity decreased from 100 nm to 150 nm”.

## Commercially available EV reference materials for standardization of EV analysis techniques

Several efforts are being undertaken to develop reference materials as EV mimetics for use as validation of assays and quality controls [34–37]. Some come in the form of synthetic materials meant to mimic biological materials, such as hollow organosilica beads and liposomes, while others are derived from biological sources [34,35,37,38]. Synthetic materials such as hollow beads have the benefit of utilizing characteristics of EVs with fine control, such as low refractive indices and a core-shell structure, whilst being in the format of a tightly defined population which will likely be unambiguous in its detectability using optical methods. Synthetic standards such as these are likely much easier to develop into certified reference materials due to being stable, homogeneous populations that are more amenable to analysis and assigning traceable metrics. However, hollow silica beads have not yet been developed to display or contain proteins or other molecules that would be useful as positive controls for assays and testing of reagents.

Biological reference materials that have been proposed in the literature include virus particles, cell culture-derived (including engineered) EVs, bacterial outer membrane vesicles and biofluid-derived EVs (from urine, plasma, serum, etc.) [38]. Each of these biological reference material types has strengths and limitations that are application dependent. The generation of biological reference materials to a standard that allows certification is challenged by the large number of parameters involved and the lack of sensitive instrumentation that can provide traceable measurements, as previously outlined. Perhaps the most achievable goal is that these EV reference materials be developed and reported in calibrated units, with uncertainity and a statement of the detection range of the reported metrics. Efforts to produce high-quality biological reference materials with calibrated measurements are recent, and have been demonstrated in the form of non-pathogenic (engineered or inactivated) enveloped virus particles or virus-like particles/recombinant EVs [34,35].

The development and validation of assays, reagents and detection methods strongly require that biological reference materials characterized with traceable measurements are commercially available. The lack of commercial availability of traceably characterized biological reference materials is in part due to the lack of calibration and reporting standards within the field. The traceability and rigour with which currently available reference materials are characterized with respect to refractive index, epitope abundance, size and concentration are shown in Table 2. Recently, an inter-societal flow cytometry working group with members from ISEV, ISAC and ISTH made an effort to overcome the lack of experimental and reporting standards for flow cytometry. This effort resulted in the MIFlowCyt-EV reporting framework, which was published as a position paper in the *Journal of Extracellular Vesicle* [15]. The majority of the MIFlowCyt-EV framework is applicable to most optical analysis techniques, despite being developed for flow cytometry. Utilization of the framework would result in a large step forward for the field, not only in standardizing reporting and being able to validate findings, but also in starting to characterize and create commercially available biological reference materials with traceable measurements.

A comparison of commercially available EV reference materials and the level to which they are characterized is collated into Table 2. The criteria from this table are metrics that we would propose as a bare minimum when considering the use of these materials for downstream characterization using an optical characterization method and reporting the results. These available reference materials include EVs, EV-sized retroviruses and liposomes. An absolute minimum of reporting EV studies is describing the method used to separate the EVs [1]. At this time, no commercial sources of biological reference materials in Table 2 provide proof of purity or indication of purification method. These reagents may, therefore, contain soluble proteins, free nucleic acids, or other co-isolates, that could have effects on the vendor or user’s downstream assays if used without further purification. The reporting of size distribution is a common and important factor when using reference materials for the characterization or validation of an assay using an optical analysis technique. While most studies report a diameter statistic, such as mean or median, few provide the variance of this diameter distribution, and no manufacturer currently provides the limit of sensitivity for the technique quantifying the diameter. This is a particular concern for polydisperse reference materials with biological derivations, such as EVs, that have been quantitated with techniques known to have a limited abilited to detect the full EV population. This is less of a concern for monodisperse reference materials, such as beads, quantitated with high-resolution techniques such as electron microscopy or small-angle x-ray scattering whose diameters are provided in traceable units. As seen in Table 2, despite limitations in sensitivity quantification and standardization (Table 1), many of the diameter distributions of currently available biological EV mimetics utilize NTA. An approximate refractive index is reported for three reference materials, two of which are viral particles. Buoyant density was provided by two manufacturers. Two manufactures provide known surface proteins and their brightness in calibrated units, while another with an extensive range of EV types gives no indication of known surface markers. Currently, no commercially available reference materials intended for use as EV references materials provide the limit of sensitivity for their measurement metrics, although two vendors do share their characterisation data in standard, but non-traceable, units.

## Development of new reference materials for standardization of frequently studied EV characteristics

### Refractive index determination

**Figure 5.**
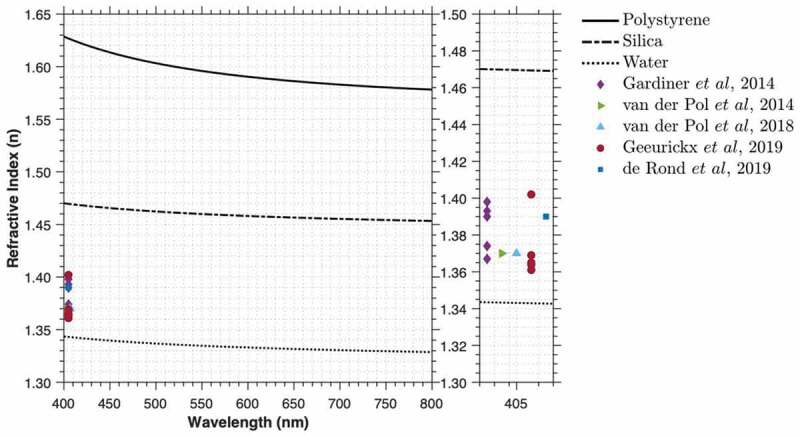
**Dispersion properties of polystyrene, silica and water from wavelengths of 400–800 nm**. Dispersion properties of polystyrene, silica and water were calculated using the Sellmeier equations for published materials [53–55]. Median refractive index (geometric mean in case of Gardiner *et al*) measurements for different EV sources were acquired from the literature [22,23,34,40,41].

The refractive index contrast between a particle of a certain material and its surrounding environment determines how much light is scattered from it [19]. The refractive index has no effect on measurements from non-optical techniques such as RPS [25]. For optical techniques that detect light scatter (e.g. flow cytometry, NTA, DLS, SP-IRIS), the refractive index strongly influences the particle measurements in the detectability of the particle or derivation of the particle diameter [19,39]. The refractive index is therefore an important metric to be provided with a reference material if it is intended for use with optical analysis techniques. Current literature suggests that while the refractive index of EVs is lower than reference materials such as silica, it is variable, Figure 5 [22,23,34,40]. Indeed, the effective refractive index of EVs will never be a single number due to an EV being a core-shell model, where the ratio of the shell (membrane) to the core (cytosolic portion) increases as EV size decreases. Smaller EVs will therefore have larger effective refractive indicies than larger EVs, such that refractive index cannot be reported with a single metric [22,23]. The term effective refractive index refers to a solid spherical particle with a given refractive index that scatters the same amount of light towards the detector as a similar-sized EV.

Calibration of optical analysis equipment for refractive index approximation generally requires utilizing particle scatter physics in the form of Mie theory [22,23,33,40,41]. While refractometers exist, they tend to be designed for liquids or films and are therefore not optimized for single particles or polydisperse mixtures of spheres. The application of Mie theory modelling requires calibration with spherical particles of known diameter and refractive index in order to fit predicted scattering models to detection equipment [22,32,35]. Many certified polystyrene reference particles exist. These are monodisperse and have low variance and a reported, but non-traceable, refractive index. For some materials, such as silica, refractive index is assumed based on published literature from measurements of silica films [42,43]. These materials may thus be variable in composition quality, with few certified particle reference materials available. The refractive indices currently provided with certified reference particles tend to be reported to two decimal places and with no indication of variance; they are therefore not a traceable metric.

Assay validation for sensitivity to detect particles of low refractive index remains difficult. Analyses of polystyrene particles were initially used to validate the sensitivity of optical analysis instrumentation [44]. However, it was soon recognized that polystyrene, having a much higher refractive index, scatters far more light than most biological particles, and therefore is not an appropriate EV mimetic [32]. Since then, focus has been directed to the use of silica particles, which, while lower in refractive index than polystyrene, still scatter more light than similarly sized EVs, Figure 5. Liposomes have more recently been suggested as a better material for validating equipment sensitivity due to the similarity in refractive index to EVs; however, it is difficult to produce monodisperse, stable liposomes with low variance at small diameters [45]. Care must be taken when preparing liposomes as extrusion methods, such as filters, can create multilamellar liposomes, thus increasing refractive index and not being representative of EVs with a single phospholipid bilayer [46].

The ability to calibrate an instrument’s axis for refractive index will however continue to require the use of light scatter modelling and not be a traceable measurement until certified traceable refractive index reference particles exist. Without the ability to compare or validate refractive index measurements to a certified reference material, the accuracy of reported measurements will remain unknown.

### Epitope measurement

Phenotyping, defined for our purposes here as classifying EVs by presence of surface markers, is a powerful tool in determining cell derivation and function of EVs. Methods to phenotype EVs vary from qualitative (e.g. Western blots), to semi-quantitative (e.g. ELISA, bead-capture assays, fluorescent NTA, EM), to quantitative (e.g. super-resolution microscopy, flow cytometry). Reference materials that display a known quantity of a particular epitope (i.e. target of an affinity reagent such as an antibody, peptide, or aptamer) will therefore be important for a wide variety of assays and analysis platforms.

#### Crucial: limit of sensitivity

As for other types of measurements, it is critical to know the limit of sensitivity of phenotyping assays. To achieve a quantitative assay, this limit should be reported in standard units so that (for example) amount of bound fluorescent antibody can be expressed in molecules of equivalent soluble fluorophore. Reporting data in quantitative units (e.g. molecules of equivalent soluble fluorophore, MESF) instead of as relative expression or in arbitrary units (fluorescence intensity) allows for comparison of data across scientific institutions and platforms [29,39]. Where this is impossible, many assays are still able to indicate the relative amount of an epitope, comparing single EVs or EV samples. In cases for which an epitope is undetectable, a lack of detection should not be confused with proof that an epitope is truly absent from an EV. Instead, the result should be reported in context of the limit of sensitivity, along with other relevant variables of the particular assay.

#### Fluorescence calibration

Development of reference materials for calibration of epitope abundance is contingent on the analysis technique. Currently, optical techniques are most widely used, relying predominantly on fluorescence for the quantification of phenotype, so fluorescence calibrators are required in the form of molecules of equivalent soluble fluorophore (MESF). These currently exist only in the form of uncertified reference beads in the range of 2–7 µm in diameter that lack traceability [26,28,29,47]. The size and fluorescence of these beads reflect their originally intended use in cellular analyses and requires extrapolation of their values (i.e. 1,000 MESF) to be applicable to the majority of EVs (i.e. <100 MESF). While the trueness of fluorescence extrapolation from bright fluorescence reference beads to dim signals has not been validated, initial small particle calibration studies extrapolating bright reference beads to dim biological signals suggest that they are precise and produce concordant data irrespective of instrumentation [29,47,48]. This is entirely expected, as the measured fluorescence intensity scales linearly with the number of molecules present. The use of fluorescence calibration is highly recommended by the ISEV-ISAC-ISTH working group for flow cytometry, as outlined in the MIFlowCyt-EV framework [15].

### Size distribution

**Figure 6.**
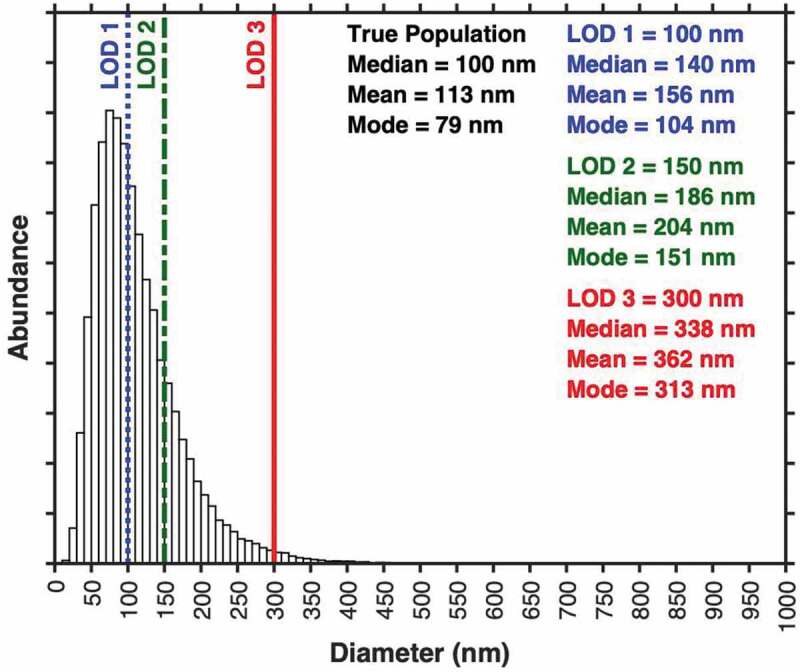
**Limitations of statistical metrics on partially resolved populations**. Shown is a hypothetical dataset with log-normal diameter distribution. Three limits of sensitivity (100 nm, blue; 150 nm, green; 300 nm, red) are shown. The summary statistics for events above these limits of sensitivity are shown in the corresponding colours in the right of the plot.

The size (diameter, radius) of EVs is one of their defining characteristics. Current evidence suggests that EVs have a log-normal diameter distribution, Figure 6 [6,18,27]. That is, abundance scales inversely with size up to the peak of the distribution at a small size, which is itself determined by biophysical characteristics of lipid bilayer-delimited particles. Currently, no high-throughput analysis method is capable of sizing the full range of EVs (Table 1) [6]. A large limitation of the current literature is that reported EV diameters or diameter distributions do not state the limit of sensitivity of the detection equipment. For example, it is often stated that EV populations have a mean diameter of 100 nm. This is a distorted perception of the true EV size distribution, as in many cases the majority of EVs (small EVs) are undetectable and thus excluded from the distribution. A single metric to describe size distribution of EV samples – such as mean, mode, median or percentile – is insufficient since it is biased by the sensitivity limit of the instrumentation, Figure 6. Techniques that rely on the composition of EVs (i.e. refractive index) for detection and inference of diameter, such as flow cytometry, NTA, DLS and interferometry, will be biased by the refractive index of individual particles. In addition, the illumination wavelength also contributes to the sensitivity of optical techniques. An NTA instrument with a 405 nm laser will produce a slightly different size distribution compared with an instrument with a 640 nm laser due to the differences in the light scattering efficiency and sensitivity of the instrument. For these reasons, optical methods require calibration in order to determine their limit of sensitivity.

#### Flow cytometry: light scatter and fluorescence

The approximation of particle diameter from light scatter or fluorescence intensity in flow cytometry requires the use of light scatter calibration and fluorescence calibration, respectively [26,32,35]. Certified size standards are available for various sizes and compositions. These certified size standards are homogeneous and traceable in their diameter measurement, made from synthetic materials providing stability, and are often reported with a density and refractive index. Flow cytometer light scatter calibration can utilize certified diameter standards for the approximation of particle diameter. This measurement uses both the diameter of the certified reference particle and the non-traceable refractive index measurement. The accuracy of diameter approximated using flow cytometer light scatter is therefore multi-factorial. Fluorescence calibration for EV size inference has been demonstrated with a liposome reference material of known diameter that is labelled with the same intercalating membrane dye as a population of EVs [26]. The fluorescence diameter measurement is also multifactorial and relies upon the accuracy of the liposome diameter, the detectability of the liposome population, and the fluorescence intensity of the liposome population as compared with the EV population. Importantly, both sizing methods allow for quantifying the limit of sensitivity in a standard unit of measurement irrespective of the instrument, allowing platform-independent comparisons.

#### NTA: considerations and limitations

NTA size particles not by a single intensity measurement, as in flow cytometry, but rather by tracking the Brownian motion of particles (multiple measurements) [21]. Size is then inferred from the Stokes-Einstein equation. NTA does, however, rely on optical intensity to track particles over a sufficient length of time to derive an accurate size. Determining the limit of sensitivity for NTA would therefore require light scatter modelling or fluorescence calibration, depending on tracking mode, as well as some way to account for: (1) the movement of particles in and out of the field of view, (2) changing intensities and (3) the ability of the instrument to track them. In light scatter mode, intensity depends on refractive index and illumination wavelength. The enumeration of particles is then affected by the camera’s varying noise, fluctuating at a pixel level over time, over which the software must identify and track a particle over several time frames. In fluorescence mode, additional information includes the amount of dye and rate of photobleaching. Currently, there is no demonstrated method to express limit of sensitivity for NTA in standard units, irrespective of factors such as refractive index of fluorescence intensity, such that comparisons could be made across instrumentation.

In summary, certified reference materials for size measurements by platforms like flow cytometry and NTA do not actually rely, or solely rely, on the physical size of the reference material. Certified reference materials that cater to calibrated fluorescence measurements and refractive index are therefore required. Currently, certified sizing standards are most applicable to calibration of non-optical methods, such as RPS, allowing for statements about accuracy and limit of sensitivity. RPS is thus a useful orthogonal technique to assess optical sizing. Similarly, cryo-electron microscopy and similar techniques, despite being low-throughput, can be used to assess EV morphology across the full range of sizes and correlate findings between detection methods.

The development of standard EV reference materials that can be used across optical techniques for validation of size distributions using light scatter requires knowledge of refractive index, which is not fixed across EV diameter and is not currently a traceable measurement using available methods. Similar standard reference materials for fluorescence require knowledge of fluorescence intensity in standard units, which, while feasible, is not yet a traceable measurement and is currently only compatible with flow cytometers that are mostly unable to detect the whole EV population. A potential method of attempting to standardize optical size measurements is under investigation. A European metrology project (METVES II) attempts to make assay validators using hollow organosilica spheres of known size that mimic the core-shell structure of EVs, **Table 2**. This project aims to help standardize the field by using EV light scatter and fluorescence mimetics to validate instrument sensitivity.

### Concentration measurement

Particle concentration may be a useful parameter to normalize EV input into an *in vitro* or *in vivo* assay, or even in the diagnostic setting. Despite being reported in almost every EV publication, EV concentration is one of the most difficult metrics to derive due to the systematic and random errors that are involved. No high-throughput analysis method has demonstrated detecting all EVs with a single configuration, Table 1. Importantly, the limit of sensitivity for techniques such as flow cytometry, NTA and RPS is generally not reported, even though this cut-off is crucial to knowing how many of the smallest and most abundant EVs are detected. The reported concentration of EVs is therefore likely one of the least accurate metrics in the literature and has been shown to increase over time [49]. While certified reference materials for concentration measurements already exist, an accurate concentration measurement of a sample requires detection of all particles. This is not the currently possible for techniques such as RPS, NTA and flow cytometry. The solution to this problem is, however, relatively simple: reporting detectable events within a given detection window, using a calibrated instrument. If an instrument has been calibrated, a concentration can be reported within a given detection window, e.g. “1.3x10^9^ particles per mL were detected between 80 and 300 nm”.

Calibrating EV analysis equipment to determine what can and cannot be detected in standard units is easier on some instruments than others. Instruments using RPS can be calibrated with size standards irrespective of optical characteristics such as refractive index. Optical techniques such as flow cytometry and NTA require light scatter calibration and/or fluorescence calibration to define their limits of sensitivity and their detection window. Both flow cytometry and NTA have a number of variables to account for, and well defined, and ideally certified reference materials would be used for their calibration. These limitations are the same as those discussed previously for epitope measurement and size distribution.

The development of EV reference materials with a known concentration is, therefore, heavily dependent on the instrumentation being used to quantify concentration and whether those instruments are capable of detecting the whole population. When they are not capable of detecting the whole population, the concentration should be reported within a defined size range of the EV population. Given that current high-throughput methods are unable to detect the full EV population, it is unlikely that an accurate concentration measurement for the full EV population can be reported at this stage for current commercially available reference materials. If the limits of sensitivity are reported with reference materials, the concentration measurement can still, however, be normalized across instrumentation.

## Discussion

The characterization of reliable EV reference materials requires the calibration of measurements obtained from any EV analysis technique. Calibration is also critical for the characterization of samples reported in published data. The utilization of current reference materials and development of new reference materials relies upon several factors. These include: (1) continuing efforts in the EV field to develop educational resources and workshops for understanding and teaching the need and utilization of standardization procedures, (2) journals enforcing minimal criteria for reporting experiments using EV analysis techniques and (3) encouraging industry to utilize and develop robust reference materials for calibration and quality control of EVs.

The development of platform-independent reference materials is needed to facilitate cross-platform standardization. While flow cytometry and NTA were a particular focus of this piece, emerging optical techniques such as super-resolution microscopy and interferometry are also becoming more widely used. The standardization of these analysis techniques will be aided by the development of reference materials for flow cytometry and NTA, since each of these techniques measures optical signals.

While the EV field currently and severely lacks standardization, this problem is recognized by the field. An example of an initiative aimed at development of traceable reference materials is METVES II, described in Text Box I. The International Society for Extracellular Vesicles (ISEV) in 2019 initiated a “Rigor and Standardization” subcommittee to coordinate task forces across several pertinent areas, one of which is “EV Reference Materials”. Recently, an ISEV workshop, hosted in Ghent, Belgium was dedicated to Rigour and Standardization. Initiatives to standardize reporting have also been made in the form of MISEV, EV-TRACK and MIFlowCyt-EV. Progress towards standardization is thus gaining momentum via concerted efforts.

**Textbox** “METVES II (https://www.metves.eu), is a European metrology project in which metrology institutes, companies and academia collaborate to achieve standardization of concentration measurements of EVs in clinical samples, such as plasma and urine [50]. METVES II focuses on standardization of EV concentration measurements by flow cytometry, since it is already available in hospitals and can characterize single EVs at high throughput (thousands/sec). All aspects of flow cytometry, including flow rate, fluorescence and light scatter (size, refractive index) need to be calibrated to produce reliable and reproducible results. Towards this goal, dedicated and traceable reference materials are being developed that combine physical properties resembling those of EVs. These materials will include stable particles with: (1) diameters between 50 nm and 1,000 nm, (2) concentrations in the range of 10^9^–10^12^ particles mL^−1^, (3) a visible fluorescence intensity between 100 and 100,000 molecules of fluorophore, and (4) a refractive index in the range of 1.37–1.42. Three types of reference materials will be developed: hollow organosilica beads (HOBs) [37], monodisperse liposomes and low-refractive-index solid particles. The size and concentration of the developed reference particles will be traceably characterized in SI units [51,52]. It is aimed that this project will deliver a single reference material to calibrate all relevant properties involved in EV flow cytometry measurements”.
